# Geographical Variation in Health-Related Physical Fitness and Body Composition among Chilean 8th Graders: A Nationally Representative Cross-Sectional Study

**DOI:** 10.1371/journal.pone.0108053

**Published:** 2014-09-25

**Authors:** Michael D. Garber, Marcelo Sajuria, Felipe Lobelo

**Affiliations:** 1 Department of Epidemiology, Emory University Rollins School of Public Health, Atlanta, Georgia, United States of America; 2 Health and Sciences Unit, Chile National Institute of Sport, Santiago, Chile; 3 Hubert Department of Global Health, Emory University Rollins School of Public Health, Atlanta, Georgia, United States of America; Instituto de Higiene e Medicina Tropical, Portugal

## Abstract

**Purpose:**

In addition to excess adiposity, low cardiorespiratory fitness (CRF) and low musculoskeletal fitness (MSF) are important independent risk factors for future cardio-metabolic disease in adolescents, yet global fitness surveillance in adolescents is poor. The objective of this study was to describe and investigate geographical variation in levels of health-related physical fitness, including CRF, MSF, body mass index (BMI), and waist circumference (WC) in Chilean 8th graders.

**Methods:**

This cross-sectional study was based on a population-based, representative sample of 19,929 8th graders (median age = 14 years) in the 2011 National Physical Education Survey from Chile. CRF was assessed with the 20-meter shuttle run test, MSF with standing broad jump, and body composition with BMI and WC. Data were classified according to health-related standards. Prevalence of levels of health-related physical fitness was mapped for each of the four variables, and geographical variation was explored at the country level by region and in the Santiago Metropolitan Area by municipality.

**Results:**

Girls had significantly higher prevalence of unhealthy CRF, MSF, and BMI than boys (p<0.05). Overall, 26% of boys and 55% of girls had unhealthy CRF, 29% of boys and 35% of girls had unhealthy MSF, 29% of boys and 44% of girls had unhealthy BMI, and 31% of adolescents had unhealthy WC. High prevalence of unhealthy fitness levels concentrates in the northern and middle regions of the country and in the North and Southwest sectors for the Santiago Metropolitan Area.

**Conclusion:**

Prevalence of unhealthy CRF, MSF, and BMI is relatively high among Chilean 8th graders, especially in girls, when compared with global estimates. Identification of geographical regions and municipalities with high prevalence of unhealthy physical fitness presents opportunity for targeted intervention.

## Introduction

In adolescents, low cardiorespiratory fitness (CRF) and low musculoskeletal fitness (MSF) are independently associated with an elevated cardio-metabolic risk profile [1]–[3]. Moreover, male adolescents with poor MSF had increased risk of all-cause premature mortality [4]. Cardio-metabolic risk factors, including CRF and MSF, track moderately into early adulthood [5], [6], although less is known about the stability of these indicators into later adulthood, where the onset of and mortality attributable to non-communicable diseases (NCDs), such as cardiovascular disease (CVD), diabetes, and cancer, occur most frequently [7]. Nonetheless, low CRF and low MSF independently predict higher risk of CVD and all-cause mortality across various adult age groups [8]–[10]. Despite strong evidence linking youth physical fitness to NCD risk [1], surveillance of this health marker is relatively poor [11], particularly beyond North America [12], [13], Europe [14], and Australia [15]. Population-based monitoring of MSF is especially deficient, compared with CRF [16].

Physical activity (PA) has been identified as a priority objective for global action in the prevention and control of NCDs [17], with an estimated 80% of youth worldwide failing to meet recommended PA guidelines [18]. Although physical fitness has an important genetic component [19], recent levels of PA are its most important modifiable determinant [20]. Therefore, improved surveillance of physical fitness can enhance the global NCD monitoring framework, providing an objective indicator linked to PA and health among youth.

In response to the global deficiency in physical fitness surveillance [21], updated guidelines for the population-based assessment of health-related fitness in youth have recently been released [22], [23]. These two fitness test protocols emphasize the importance of assessing health-related rather than performance-based physical fitness and reinforce a critical need for wide implementation. Surveillance is particularly important in Latin America and other regions outside of Europe and the U.S., where the burden of NCDs and their risk factors are rapidly increasing [7].

Despite the well-documented epidemiologic and nutritional transition fueling the NCD epidemic in Latin America [24], limited local data are available to guide NCD prevention programs and policies, in particular around low PA and fitness [25]. In Chile, a country where the epidemiologic transition is well-established [26], youth obesity and PA have been added to monitoring systems by the government [27] and research institutions [26]. In 2010, health and education authorities in Chile added a physical fitness assessment to the national education survey [28]. To the best of our knowledge, until this point, neither CRF nor MSF had been assessed at the population level in Chile, or anywhere else in Latin America, in any age group. The objective of the present study is to present nationally representative estimates of health-related physical fitness, including CRF, MSF, and body composition, from a large, population-based sample of Chilean 8th graders and investigate potential geographical variations in their prevalence.

## Methods

### Study sample and design

This cross-sectional study is based on data from a nationally representative sample of 8th-grade students (median age = 14.1 years; 25th-75th percentile: 13.8–14.4 years) assessed in the Chilean National Physical Education Survey, which was administered by the Chilean Ministry of Education (MINEDUC) in November of 2011 [29]. The data are available upon request here: http://www.agenciaeducacion.cl/investigadores/bases-de-datos-nacionales/(accessed 1 Jul 2014). The proportionally allocated sample was stratified by 15 regions and three school types. Within each stratum, schools were the primary sampling unit, and all students in selected schools were sampled. Between 93% and 99% of Chilean 8th-grade-age students matriculate in school [30].

A total of 28,649 students were considered for physical fitness evaluation from all regions and 227 municipalities (“comunas”) of Chile [29]. Due to erroneous data entry, student disability, student temporary illness or injury, student chronic illness, or student absenteeism [29], MINEDUC limited the sample to 19,929 students. We then excluded students 18 years and older or with missing body mass index (BMI) measurement for a sample of 19,904 students. Of that sample, some students (n = 1,976) were excluded from the 20-meter shuttle run CRF test (20mSRT) based on performance in a screening test in which participants walked at a constant pace around a 50-meter course. If, at the end of three minutes, their heart rate exceeded 160 beats per minute, they were deemed ineligible to participate in the 20mSRT [29]. This screening criterion resulted in a final working sample of 17,928 students for the 20mSRT, corresponding to 63% of the original sample and 90% of the working sample.

### Physical fitness assessment

Consistent with recommendations [22], [23], we limited our analysis to health-related [3], valid [31], and reliable [32] field-based tests to assess three dimensions of physical fitness: the 20mSRT to estimate maximal oxygen consumption (VO2max), the gold-standard measurement for CRF [33]; standing broad jump (SBJ), to assess MSF; and BMI and waist circumference (WC) to assess body composition. Tests to estimate CRF, MSF, and body composition in adolescents have strong predictive validity to discriminate cardio-metabolic risk [2], [3], [34]. Strong evidence indicates that, in youth, the 20mSRT is a valid test of CRF [31], the SBJ can be considered a general index of lower (R^2^ = 0.829–0.864) -and-upper (R^2^ = 0.694–0.851) -body MSF [35], and BMI and WC are good estimates of body composition [31]. Three of the four field tests have shown high levels of reliability in adolescents in the school setting (20mSRT, SBJ, BMI: no inter-trial difference; WC: significant inter-trial difference) [32].

Testing procedures were consistent with guidelines for school-based fitness assessment [36]. At each school, a team of trained MINEDUC evaluators administered the tests in partnership with the physical education instructor. Tests occurred in the school gymnasium or another hard surface available [29]. The 20mSRT was administered as described by Leger et al [37]. Participants ran in a straight line between two lines 20 m apart, while keeping pace with pre-recorded audio signals. The initial speed was 8.5 km/hour and increased by 0.5 km/hour per minute. The test was finished when the participant failed to reach the end lines keeping pace with the audio signals on two consecutive occasions or when the subject stopped because of fatigue. Results were recorded to the nearest stage (minute) completed. In the SBJ, students jumped as far as possible standing with feet shoulder-width apart. The farthest of two scores was recorded, to the nearest 0.1 cm, as the distance between toes at take-off and heels at landing, or whichever body part landed nearest to take-off [29]. Weight was measured to the nearest 0.1 kg [29]. Height was measured to the nearest 0.1 cm [29]. WC was measured by horizontally placing an inelastic tape measure midway between the lowest rib margin and the iliac crest to the nearest 0.1 cm. During the anthropometric measurements, students wore light clothing and were barefoot. Data were recorded on paper by the MINEDUC evaluators [29]. As part of the same evaluation, students performed three tests not analyzed in this study: push-ups and sit-ups to assess MSF, and sit-and-reach to assess flexibility [29].

### Classification of health-related fitness

Rather than relying on performance-based normative values, we categorized physical fitness data using cut-points shown to have cardio-metabolic health predictive value [38], [39]. This criterion-referenced system can be considered a valid approach for the study of physical fitness in the context of NCD prevention [3]. To estimate VO2max using the 20mSRT, the equation developed by Leger et al [37] was used. To classify VO2max into health-related categories, we used the 2011 FITNESSGRAM standards, which categorize children into three groups: healthy; needs improvement (NI); and NI-health risk [39]. These age-and-sex-specific VO2max cut-points were validated against the presence of metabolic syndrome using nationally representative U.S. data [39]. Criterion-referenced cut-points for MSF, assessed via the SBJ, have not been explicitly established. In two studies, the least fit quartile showed the strongest association with a poor cardio-metabolic risk profile [40], [41], suggesting this could be considered the unhealthy group. Based on the mentioned approach, we conservatively considered the unhealthy MSF group to be the age-and-sex-specific 20th percentile reported for European adolescents [14]. For BMI, the 2011 FITNESSGRAM standards [42] were explicitly developed with health outcomes in mind. Therefore, we used these cut-points for most analyses, but also present the World Health Organization 2007 [43] and the Cole et al. International Obesity Task Force (IOTF) [44] cut-points for comparative purposes, as has been previously recommended [45]. To classify WC, we used criterion-referenced health-related cut-points derived from a U.S representative sample [46] because of its large sample size, age-specificity, and relatively generalizable ethnicity. Comunas were classified as either urban or rural according to the method proposed by Berdegue et al [47]. A total of 46 of the 52 comunas in the Santiago Metropolitan Region had fitness data. These comunas were grouped into seven commonly used geographical sectors for the Santiago Metropolitan Area [48].

### Statistical analysis

Normality for selected variables was verified using histograms and Q-Q plots. Prevalence estimates are weighted within stratum. Standard errors were calculated using the Taylor series linearization method. Equality of sex proportion in the sample was assessed with a design-based goodness of fit test. Chi-square was used to compare dichotomous variable frequencies across sex categories. For variables with greater than two categories, logistic regression was used to test differences in sex proportion within category. T-tests were used to compare fitness variable means across sexes. In addition to assessing the prevalence of the health-risk group of each fitness variable individually, we investigated prevalence of health-risk for different combinations of fitness variables (e.g. prevalence of needs improvement – health risk CRF combined with unhealthy MSF). To investigate potential for internal selection bias in CRF, MSF, or urban vs. rural, we calculated the relative prevalence of a missing value by other demographic and fitness variables using logistic regression [49]. Missings in BMI and WC were not evaluated due to very few missing values in the working sample (BMI: n = 0; WC: n = 9).

We created chloropleth maps to show the prevalence of unhealthy physical fitness at the country level, by region, and in the Santiago Metropolitan Region, by sector, for each of the four fitness variables. For CRF and BMI, the unhealthy group on the maps represents the combined NI and NI-health-risk FITNESSGRAM groups [39], [42]. MSF and WC follow the aforementioned dichotomous categorization. On each map, prevalence of unhealthy fitness was classified into four categories, compromising between quantile and equal interval methods [50]. Color selection was guided by colorbrewer2.org.

We further investigated geographical variation for each of the four fitness variables. Logistic regression was used to identify the relative prevalence of unhealthy physical fitness in each of the 14 regions, compared with the referent Santiago Metropolitan Region [49]. An interaction term identified the relative prevalence of unhealthy physical fitness in urban versus rural areas by region (Santiago vs. all others). Logistic regression was also used to identify the relative prevalence of unhealthy physical fitness in each of six geographical sectors in the Santiago Metropolitan Region, compared with the referent Northeast sector. Prevalence ratios were favored to odds ratios due to the high prevalence of unhealthy physical fitness [51]. Crude prevalence ratios are presented to reflect true geographical distribution. Analyses were performed in SAS-callable SUDAAN [52] to account for the complex sampling design. Maps were created in ArcGIS [53]. Statistical significance was determined at p<0.05.

### Consent and ethical approval

The National Physical Education Survey was authorized under the Chilean Law of Sport number 19.712, article 5 [29]. MINEDUC solicited consent from schools prior to testing and instructed each school to inform parents and students with a standardized script about the nature and importance of the fitness tests, the assessment date and time, and how to prepare for the tests [29]. Students with temporary illness or injury, special needs, physical disabilities, or chronic illness were exempt to take the test if they presented a doctor’s or parent’s note. MINEDUC also requested an emergency plan from the school in the event of an adverse event. The study authors entered a written data use agreement with MINEDUC. As it was a secondary analysis of de-identified data, Emory University Institutional Review Board considered the study exempt of review.

## Results

### Descriptive characteristics

The descriptive characteristics of the Chilean 8th-grade students are presented in Table 1. Roughly half of the students were 14 years old, most attended public or subsidized schools, and about two thirds lived in urban areas. According to both the WHO and IOTF BMI cut-points, more girls were overweight than boys (30.5% vs. 26.0%; p<0.0001 and 28.5% vs. 24.2%; p<0.0001, respectively). The IOTF cut-points systematically classified fewer students as overweight or obese compared with the WHO cut-points.

**Table 1:** Demographic characteristics, including body mass index, of a representative sample (N = 19,904) of Chilean 8th-grade students: The 2011 National Physical Education Survey.

Characteristic	Males (n = 10,309)			Females (n = 9,595)			P value^†^
Total	51.8%	0.9%	10,309	48.2%	0.9%	9,595	0.04
Age (years)	14.3	0.01	10,309	14.1	0.01	9,595	<0.0001
13–13.9	36.5%	0.6%	3,775	42.6%	0.6%	4,089	<0.0001
14–14.9	49.3%	0.8%	5,086	48.9%	0.6%	4,696	0.64
15–15.9	10.5%	0.4%	1,072	6.6%	0.4%	626	<0.0001
16–17.9	3.7%	0.3%	376	1.9%	0.2%	184	<0.0001
Socioeconomic status^a^							
Low	12.6%	1.2%	1,278	11.6%	1.2%	1,100	0.23
Low-middle	32.0%	2.0%	3,276	32.2%	2.2%	3,081	0.86
Middle	34.2%	2.3%	3,469	34.6%	2.4%	3,271	0.80
Middle-high	12.5%	1.7%	1,293	14.6%	1.9%	1,406	0.14
High	8.8%	1.5%	942	7.0%	1.1%	705	0.27
School Type							
Private	8.2%	1.4%	888	6.0%	0.7%	612	0.11
Subsidized	46.5%	1.6%	4,743	48.8%	1.7%	4,623	0.19
Public	45.3%	1.5%	4,678	45.2%	1.6%	4,360	0.97
Urbanicity^b^							
Rural	36.1%	2.2%	3,265	38.1%	2.2%	3,172	0.28
Urban	63.9%	2.2%	5,710	61.9%	2.2%	5,122	
Body mass index (WHO 2007)^d^	21.8	0.05	10,309	22.7	0.05	9,595	<0.0001
Normal	61.2%	0.7%	6,299	57.8%	0.6%	5,550	<0.0001
Overweight	26.0%	0.5%	2,678	30.5%	0.5%	2,922	<0.0001
Obese	12.8%	0.4%	1,332	11.7%	0.4%	1,123	0.04
Body mass index (IOTF)^e^	21.8	0.05	10,309	22.7	0.05	9,595	<0.0001
Normal	68.2%	0.6%	7,026	63.9%	0.6%	6,135	<0.0001
Overweight	24.2%	0.5%	2,497	28.5%	0.5%	2,736	<0.0001
Obese	7.6%	0.3%	786	7.5%	0.3%	724	0.92

Data are mean (standard error) or percent (standard error) and n of category within sex. ^†^P-values reflect sex differences within strata. ^a^Groups defined by Chilean Ministry of Education [101]. ^b^Berdegue et al [47]; n, urbanicity = 17,269. ^c^WHO BMI-for-age cut-points [43]. ^d^International Obesity Task Force BMI cut-points [44].

### Physical fitness levels

Table 2 presents health-related physical fitness data by sex. Girls had twice the prevalence of NI-health-risk CRF as boys (30.6 vs. 15.0%; p<0.001), a higher prevalence of unhealthy MSF (34.7% vs. 29.4%; p<0.001), and a higher prevalence of NI-health-risk BMI (28.6% vs. 21.6%; p<0.001). The prevalence of health-risk levels of combined fitness variables was higher in girls in all combinations examined (p<0.004). For example, girls had a higher prevalence of health-risk fitness according to all four fitness variables (6.5% vs. 5.0%; p<0.001) and a higher prevalence of health-risk fitness according to at least one fitness variable (61.1% vs. 50.0%; p<0.001).

**Table 2:** Health-related physical fitness in a representative sample (N = 19,904) of Chilean 8th-grade students: The 2011 National Physical Education Survey.

Fitness characteristic	Males (n = 10,309)			Females (n = 9,595)			P value^†^
Cardiorespiratory fitness (mL/kg/min)^a^	46.4	(0.13)	9,822	39.2	(0.12)	8,106	<0.0001
Healthy	74.3%	(0.7%)	7,286	44.6%	(1.0%)	3,606	<0.0001
Needs improvement	10.8%	(0.4%)	1,063	24.8%	(0.6%)	2,012	<0.0001
NI - health risk	15.0%	(0.5%)	1,473	30.6%	(0.9%)	2,488	<0.0001
Musculoskeletal fitness (cm)^b^	169.3	(0.71)	10,240	131.1	(0.57)	9,537	<0.0001
Healthy	70.6%	(0.8%)	7,239	65.3%	(1.0%)	6,250	<0.0001
Unhealthy	29.4%	(0.8%)	3,001	34.7%	(1.0%)	3,287	
Body mass index (kg/m^2^)^c^	21.8	(0.05)	10,309	22.7	(0.05)	9,595	<0.0001
Healthy	61.2%	(0.7%)	6,303	55.7%	(0.6%)	5,348	<0.0001
Needs improvement	17.2%	(0.4%)	1,770	15.8%	(0.4%)	1,509	0.009
NI - health risk	21.6%	(0.5%)	2,236	28.6%	(0.6%)	2,738	<0.0001
Waist circumference (cm)^d^	74.3	(0.15)	10,305	71.9	(0.17)	9,590	<0.0001
Healthy	69.2%	(0.6%)	7,132	69.2%	(0.7%)	6,647	0.99
Unhealthy	30.8%	(0.6%)	3,173	30.8%	(0.7%)	2,943	
Combined fitness categories							
NI - health-risk CRF & unhealthy MSF	9.2%	(0.4%)	898	16.1%	(0.7%)	1,292	<0.0001
NI - health-risk CRF & (NI-health-risk BMI or unhealthy WC)	9.3%	(0.4%)	914	15.9%	(0.5%)	1,287	<0.0001
Unhealthy MSF & (NI-health-risk BMI or unhealthy WC)	15.1%	(0.5%)	1,546	16.9%	(0.6%)	1,607	0.004
Health risk according to all 4 fitness variables	5.0%	(0.3%)	495	6.5%	(0.3%)	521	0.0004
Health risk according to at least 1 fitness variable	50.0%	(0.8%)	5,150	61.1%	(0.9%)	5,852	<0.0001

Data are mean (standard error) or percent (standard error) and n of category within sex. NI, needs improvement; CRF, cardiorespiratory fitness; MSF, musculoskeletal fitness; BMI, body mass index; WC, waist circumference. ^†^P-values reflect sex differences within strata. Cardiorespiratory fitness is classified according to FITNESGRAM 2011 maximal aerobic capacity cut-points [39]. ^b^Unhealthy musculoskeletal fitness is standing broad jump below the 20th percentile of European adolescents [14]. ^c^Body mass index is classified according to the FITNESSGRAM 2011 health-related standards [42]. ^d^Waist circumference is classified according to health-related cut-points [46].

### Geographical variation in physical fitness

**Figure 1:**
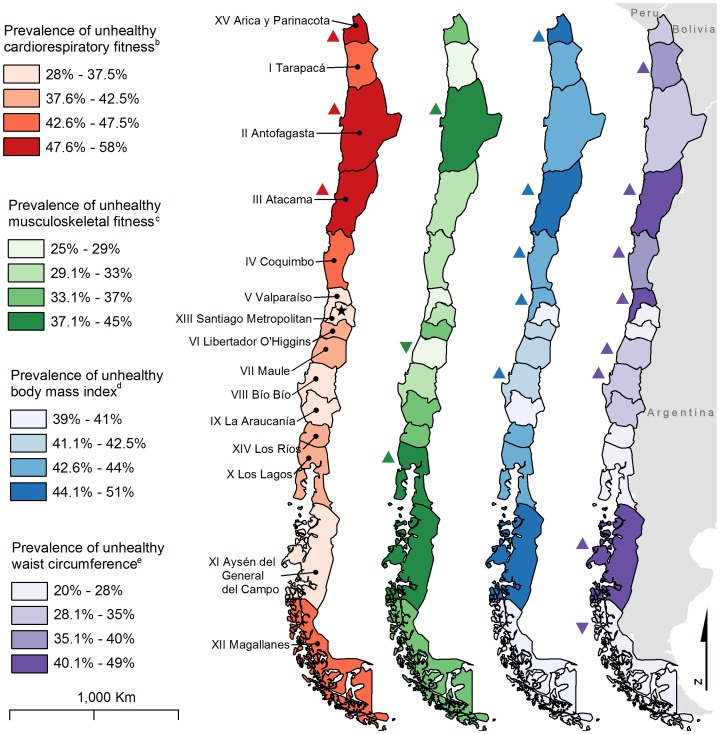
High prevalence of unhealthy physical fitness among Chilean 8th-grade students (N = 19,904)^a^ concentrates in the northern and middle regions of the country. Δ Prevalence of unhealthy physical fitness is significantly higher than in the Santiago Metropolitan Region (p<0.05). ▽ Prevalence of unhealthy physical fitness is significantly lower than in the Santiago Metropolitan Region (p<0.05). ^a^Sample size varies for each fitness variable: n, cardiorespiratory fitness = 17,928; n, musculoskeletal fitness = 19,777; n, body mass index = 19,904; n, waist circumference = 19,895. ^b^Unhealthy cardiorespiratory fitness is combined needs improvement and needs improvement – health risk FITNESGRAM 2011 maximal aerobic capacity groups [39]. ^c^Unhealthy musculoskeletal fitness is defined as standing broad jump below the 20^th^ percentile of European adolescents [14]. ^d^Unhealthy body mass index is combined needs improvement and needs improvement – health risk FITNESGRAM 2011 BMI groups [42]. ^e^Waist circumference is classified according to health-related cut-points [46].

Compared to Santiago Metropolitan, prevalence of unhealthy CRF was higher in three northern regions (all p<0.05; Figure 1). Prevalence of unhealthy MSF was higher in Antofagasta and in Los Lagos, but lower in Libertador O’Higgins (all p<0.05). Prevalence of unhealthy BMI was higher in Arica y Parinacota, Atacama, Coquimbo, Valparaíso, and Bío Bío (all p<0.05). Prevalence of unhealthy WC was higher in Tarapacá, Atacama, Coquimbo, Valparaíso, Libertador O’Higgins, Maule, and Aysén del General del Campo, but lower in Magallanes (all p<0.05).

**Figure 2:**
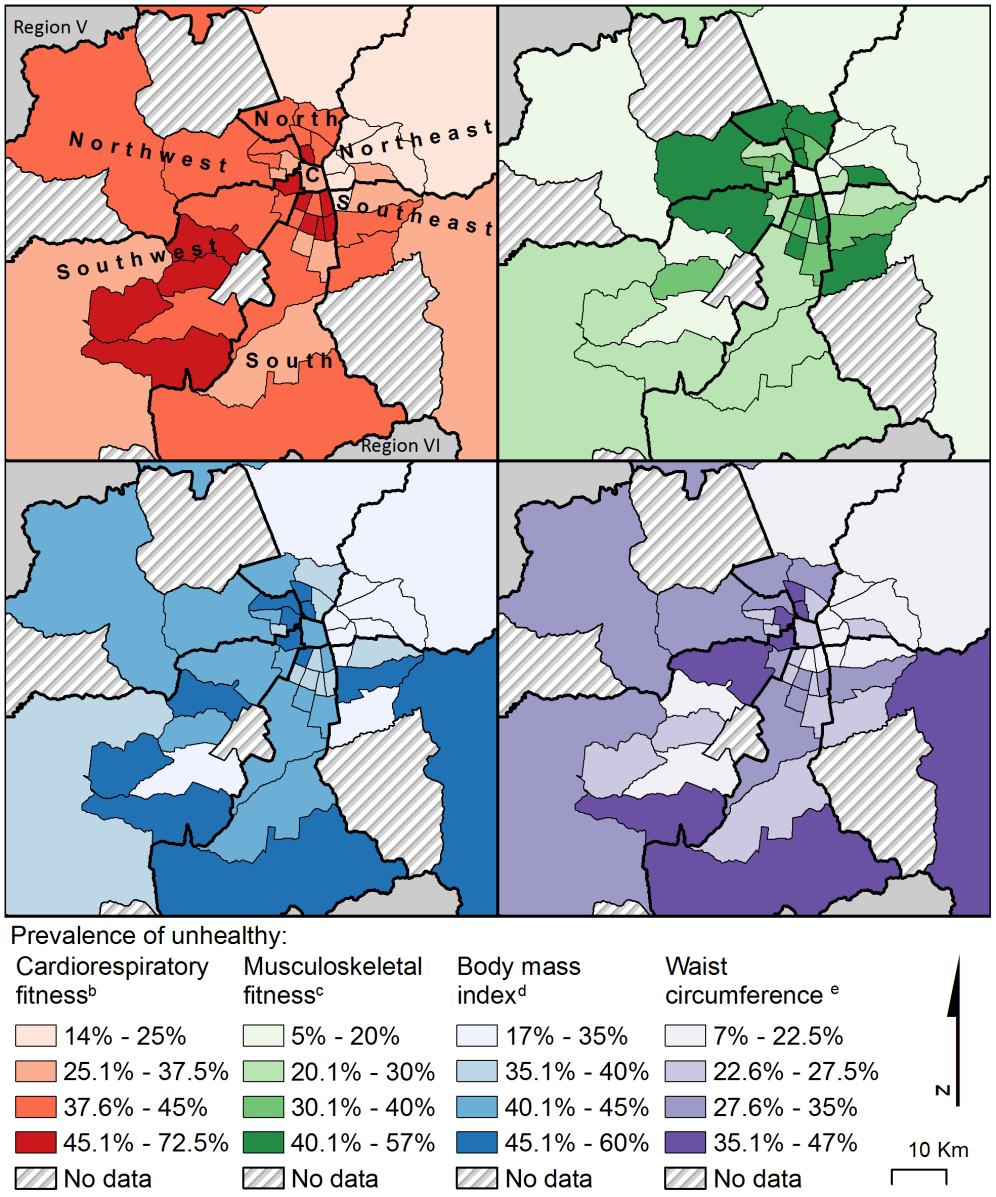
High prevalence of unhealthy physical fitness among Chilean 8th-grade students levels concentrates in the north and southwest sectors of the Santiago Metropolitan Area (n, Santiago Region = 6,957). ^a^C, Central. Comunas (municipalities) were grouped into 7 sectors based on geographical location. ^a^Sample size varies for each fitness variable: n, cardiorespiratory fitness = 6,082; n, musculoskeletal fitness = 6,894; n, body mass index and waist circumference = 6,957. ^b^Combined needs improvement and needs improvement – health risk FITNESGRAM 2011 maximal aerobic capacity groups. ^c^Unhealthy musculoskeletal fitness is defined as standing broad jump below the 20^th^ percentile of European adolescents [14]. ^d^Unhealthy body mass index is combined needs improvement and needs improvement – health risk FITNESGRAM 2011 BMI groups [42]. ^e^Waist circumference is classified according to health-related cut-points [46].

In the Santiago Metropolitan Region, in which 90.5% of students lived in urban areas (Table 3), prevalence of unhealthy MSF was higher in urban areas, but prevalence of unhealthy BMI and WC was higher in rural areas (all p<0.05; Table 3). In regions outside of Santiago Metropolitan, prevalence of unhealthy CRF was higher in urban areas (p<0.05; Table 3). Compared with the Northeast sector of Santiago Metropolitan, prevalence of unhealthy fitness for all four fitness variables was significantly higher in each of the other six sectors (all p<0.05; Figure 2 and Table 3), with the exception of MSF in the Central sector. The North and South sectors had the highest prevalence of unhealthy CRF, BMI, and WC while the North and Southeast sectors had the highest prevalence of unhealthy MSF (Figure 2 and Table 3).

**Table 3:** Geographical variation in health-related physical fitness of Chilean 8th-grade students: The 2011 National Physical Education Survey.

Geographical characteristic	Unhealthy cardio- respiratory fitness^a^		Unhealthy musculo- skeletal fitness^b^		Unhealthy body mass index^c^		Unhealthy waist circumference^d^	
	PR	(95% CI)	PR	(95% CI)	PR	(95% CI)	PR	(95% CI)
Urban^e^ (reference = rural)								
Santiago Metropolitan^f^	0.96	(0.81, 1.15)	1.46	(1.17, 1.83)*	0.88	(0.80, 0.97)*	0.80	(0.69, 0.93)*
				‡		‡		‡
All other regions	1.16	(1.06, 1.28)*	0.91	(0.81, 1.03)d	1.01	(0.96, 1.06)*	0.99	(0.91, 1.08)d
Santiago Metropolitan city sectors^g^								
Northeast (reference)	1.00		1.00		1.00		1.00	
North	2.30	(1.58, 3.33)*	3.14	(2.09, 4.71)*	1.64	(1.29, 2.08)*	1.93	(1.59, 2.35)*
Northwest	2.06	(1.38, 3.07)*	2.51	(1.59, 3.95)*	1.58	(1.24, 2.01)*	1.73	(1.38, 2.17)*
Southwest	2.33	(1.59, 3.43)*	2.33	(1.52, 3.57)*	1.62	(1.29, 2.04)*	1.93	(1.59, 2.34)*
South	2.07	(1.43, 2.99)*	2.55	(1.73, 3.77)*	1.61	(1.28, 2.03)*	1.68	(1.38, 2.05)*
Southeast	2.06	(1.42, 2.98)*	2.78	(1.81, 4.27)*	1.48	(1.16, 1.89)*	1.51	(1.21, 1.87)*
Central	1.83	(1.25, 2.68)*	1.17	(0.78, 1.77)d	1.57	(1.22, 2.01)*	1.43	(1.16, 1.77)*

PR, unadjusted prevalence ratio; CI, confidence interval. ^a^Unhealthy cardiorespiratory fitness is combined needs improvement and needs improvement – health risk FITNESGRAM 2011 maximal aerobic capacity groups [39]. ^b^Unhealthy musculoskeletal fitness is defined as standing broad jump below the 20th percentile of European adolescents [14]. ^c^Unhealthy body mass index is combined needs improvement and needs improvement – health risk FITNESGRAM 2011 BMI groups [42]. ^d^Waist circumference is classified according to health-related cut-points [46]. ^e^Berdegue et al [47]; n, urbanicity = 17,269. ^f^90.5% urban, per present definition. ^g^Comunas were grouped into 7 sectors based on geographical location. Figure 2 presents a map of the 7 sectors. Sample size in Santiago Metropolitan Region varies for each fitness variable: n, cardiorespiratory fitness = 6,082; n, musculoskeletal fitness = 6,894; n, body mass index and waist circumference = 6,957. ‡Effect of urban versus rural is different within Santiago Metropolitan Region than in all other regions (p<0.05). *p<0.05.

## Discussion

The overall goal of this study was to present nationally representative estimates of health-related cardiorespiratory and musculoskeletal fitness and body composition, of a large, representative sample of Chilean 8th graders and to explore potential geographical variation in these parameters. We found a high prevalence of unhealthy levels of physical fitness in Chilean 8th graders, especially among girls, compared with estimates of adolescents from other countries [14], [54]–[57]. Substantial geographical variation in the prevalence of unhealthy levels for all four physical fitness variables was observed, with the highest estimates concentrating in the northern and middle regions of the country and in the north and southwest sectors for the Santiago Metropolitan Area. Overall, most adolescents in this sample, five of out ten boys and six out of ten girls, were at increased health risk for at least one of the fitness components. In addition, a substantial proportion of adolescents, 5.0% of boys and 6.5% of girls, were at increased health risk all four fitness indicators combined and thus can be considered to be at an even higher risk for cardio-metabolic disease [3], [58]. These results provide an evidence-based portrayal of future NCD burden, as each fitness component assessed was selected [3], [22], [23] and classified [39], [40], [42], [46] according to its contribution to cardio-metabolic disease risk. To the best of our knowledge, this is the first population-based study to comprehensively assess the prevalence of health-related fitness for various components including body composition (BMI and WC), but most notably CRF and MSF, in any age group in Latin America. Given the availability of standardized batteries to monitor fitness [22], [23], its strong association to current and future health [3], and its value as an objective indicator of recent PA behaviors [20], surveillance of physical fitness deserves attention in the global NCD prevention agenda.

### Cardiorespiratory fitness

The prevalence of adolescents with unhealthy CRF–either needs improvement or needs improvement health risk–in the present sample, was similar to the prevalence among 7^th^ graders in the U.S. state of California [59] and among Midwestern U.S. adolescent boys, but higher than Midwestern U.S. adolescent girls [54], and higher than 14-year-olds from East England [60] (Table 4). According to the 2004 FITNESSGRAM CRF standards [61], a quarter of the present Chilean sample was in the unhealthy range for CRF, a higher prevalence than adolescents in Sweden [55] and Spain [56], but similar to adolescents from Australia [15] (Table 4). In contrast, a lower proportion of Chilean 8th graders had unhealthy CRF compared to adolescents from a Pan-European sample [14] and the U.S. [38], and boys from Bogota, Colombia [62]. Since low CRF is a strong and independent predictor for developing cardio-metabolic disease [3], [8], these data can be useful to guide primordial NCD prevention efforts in Chile [63]. Furthermore, the determination of the criterion-referenced prevalence of health-risk CRF in Chilean 8th graders enhances population-based surveillance efforts in Latin America, a region where CRF surveillance is especially weak [11], despite an alarmingly high prevalence of physical inactivity by global standards [18].

**Table 4:** Prevalence of unhealthy physical fitness among Chilean 8th graders and selected comparable^a^ studies in other countries.

				Percent unhealthy^b^	
Fitness measure	Location	Sample year	Standard	Boys	Girls
Cardiorespiratory fitness	*Chile* (R)^c^	2011	FITNESSGRAM 2011 [39]	26%	55%
	U.S. (California) [59]	2013	FITNESSGRAM 2011	28%	44%
	U.S. (Midwest) [54]	2010	FITNESSGRAM 2011	26%	23%
	England (East) [60]	2013	FITNESSGRAM 2011	12%	25%
	*Chile* (R)^c^	2011	FITNESSGRAM 2004 [61]	24%	25%
	U.S.[12] (R)	1999–2002	FITNESSGRAM 2004	35%	35%
	Colombia (Bogota) [62]	2008	FITNESSGRAM 2004	37%	–
	Sweden [55] (R)	2008	FITNESSGRAM 2004	9%	20%
	Spain [56](R)	2003	FITNESSGRAM 2004	19%	17%
	Pan-European [14]	2008	FITNESSGRAM 2004	39%	43%
	Australia [15]	1985–2009	FITNESSGRAM 2004	29%	23%
Musculoskeletal fitness	*Chile* (R)^c^	2011	20th percentile, Europe [14]	29%	35%
	Pan-European [14]	2008	20th percentile, Europe	20%	20%
	Australia [15]	1985–2009	20th percentile, Europe	<20%^d^	<20%^d^
	Norway [57]	2006	20th percentile, Europe	<20%^d^	<20%^d^
Body mass index	*Chile* (R)^c^	2011	FITNESSGRAM 2011 [42]	39%	44%
	U.S. [42] (R)	1999–2004	FITNESSGRAM 2011	17%	20%
	*Chile* (R)^c^	2011	IOTF [44]	32%	36%
	U.S. [64] (R)	1999–2004	IOTF	38%	35%
	Pan-American [65] ^d^	1988–2002	IOTF	28%	
	Canada [66] (R)	2004	IOTF	32%	26%
	*Chile* (R)^c^	2011	WHO[43]	39%	42%
	Canada [66] (R)	2004	WHO	37%	29%
	Brazil [67]	2007	WHO	20%	20%
	Spain [68] (R)	2012	WHO	17%	
Waist circumference	*Chile* (R)^c^	2011	Messiah et al. [46]	31%	31%
	U.S. [46] (R)	1999–2004	Messiah et al.	47%	48%
	Brazil [69]	2007	Taylor et al. [72]	28%	36%
	Pan-European [70]	2000	McCarthy et al. [73]	16%	19%

a Ages 12–17 years and similar fitness testing and data reporting procedures. ^b^If three categories reported, unhealthy combines the two higher-risk categories. (R) Sample is nationally representative. ^c^Present study data. ^d^Inferred by comparing published percentiles. ^e^Sample includes youth younger than 12 year old.

### Musculoskeletal fitness

The prevalence of unhealthy MSF in Chilean 8th graders was about 1.5 times that of European adolescents [14], who had yet a higher prevalence of unhealthy MSF than Australian [15] adolescents (Table 4). Although SBJ-assessed MSF has been evaluated in adolescents from other countries [16], the use of different classification approaches make further international comparison difficult. In the present study we classified unhealthy MSF levels with reference to cardio-metabolic risk [14], [40]. Despite its predictive validity in adolescents [2], [3], [34], global MSF surveillance is almost non-existent [16], particularly outside of Europe. This study adds to the international MSF surveillance efforts. The data can also be used to guide muscle-strengthening PA interventions locally for purposes of primordial NCD prevention and health promotion.

### Body mass index

In this study, the prevalence of health-risk BMI was high when contrasted with other countries, particularly in girls. Compared to U.S. adolescents, the Chilean 8th graders had over twice the prevalence of unhealthy BMI defined using the FITNESGRAM standards [42] (Table 4). On the contrary, defined using the IOTF standards [44], Chilean 8th graders had similar unhealthy BMI prevalence to a U.S. representative sample [64]. The IOTF prevalence of unhealthy BMI in Chilean 8th graders exceeds the 2006 estimate for school-age children in the Americas, which had the highest cumulative overweight and obesity prevalence of all six worldwide regions considered [65]. Based on the WHO classification [43], about 4 in 10 Chilean 8th graders were either overweight or obese, a similar prevalence to adolescents from Canada [66], but considerably higher than adolescents from Brazil [67] and Spain [68]. Based on previous estimates of obesity among adolescents in Chile [26], the high prevalence of overweight and obesity found in this study is not surprising but, nonetheless, of concern. The present study provides a baseline with regional estimates using three different classification systems, which allow for flexible international comparison and will inform future temporal trends.

### Waist circumference

In the present study, 31% of students had unhealthy WC, which is considerably less than the prevalence for U.S. adolescents [46], similar to adolescents from a Brazilian city [69], and higher than adolescents from Europe [70] (Table 4). Although approaches for classifying unhealthy WC vary greatly worldwide [71], aforementioned samples use the same [46] or similar [72], [73] standards. Waist circumference has been shown to be an independent contributor to NCD risk in youth [46]. Chilean 8th graders with abdominal obesity are thus likely at increased cardio-metabolic risk and will benefit from preventive interventions.

### Potential determinants of unhealthy physical fitness in Chile

The overall high prevalence of unhealthy fitness in this study is consistent with a growing body of evidence placing Chile at the forefront of the nutritional transition in Latin America [26], [74]. Chile’s rapid economic development and modernization has led to the “westernization” of the Chilean diet [75], characterized by an increase in the absolute number of calories, saturated fat, and fast food consumed, and a decrease in the consumption of legumes, fruit, and cereals [76]. These dietary patterns may partially explain our results, given the effect of diet on physical fitness in adolescents [77]. Concurrently, prevalence of an inactive lifestyle has increased [75]. In 2009, 21% of Chileans 15–24 years of age were physically inactive, with young women being over twice as inactive (29%) than young men (13%) [27], a sex disparity consistent with global estimates [18]. The higher prevalence of physical inactivity among young women in Chile may help explain the higher prevalence of unhealthy CRF and MSF observed in 8th grade girls, as PA is a primary determinant of physical fitness [20].

In addition, Chile has a world-leading smoking prevalence among 13–15-year-olds [78]. Among girls this age, four in ten smoke, the highest prevalence in the world [78]. The smoking epidemic could also help to explain the relatively high prevalence of unhealthy fitness in the Chilean 8th graders, in particular among girls, given the adverse effect of smoking on both CRF [79] and MSF [80]. Furthermore, this high prevalence of smoking may be an indication of a broader attitude towards wellness in Chilean youth. Adolescent smoking is inversely associated with team sports participation and general PA levels [81], which are each protective factors for unhealthy physical fitness [20], [55], including poor body composition [82]. In summary, the observed high prevalence and sex differences of unhealthy fitness in Chilean 8th graders may be explained by the behavioral and environmental risk factors to which Chilean 8th graders are currently exposed, including diet, lifestyle, and smoking, but other environmental and socio-economic correlates still need to be explored.

### Geographical variation of physical fitness

Substantial geographical heterogeneity was observed in the prevalence of unhealthy physical fitness in the Chilean 8th graders, which may be a result of geographical variation in previously mentioned risk factors in addition to geographical differences in economic development or climate. Santiago Metropolitan Region, home to over a third of Chile’s population, had lower prevalence of unhealthy physical fitness than many other regions of the country (Figure 1). This difference may be due in part to Santiago Metropolitan’s relatively high economic development compared with the rest of the country [83], as higher socioeconomic status (SES) has been linked with lower prevalence of unhealthy physical fitness and body composition in other settings [84]. Geographical variation in physical fitness may also be due in part to climactic differences across Chile, which extends across a great range of latitudes and climate zones. Cold, wet, or very hot climates have been shown to deter physical activity in adolescents [85], but this trend fails to explain higher prevalence of unhealthy fitness in the arid and warm, but not extremely hot, northern regions, such as Atacama, Arica y Parinacota, and Antofagasta. Most regions of Chile experience four distinct seasons. Seasonality may have influenced fitness, as tests were administered in November, spring in Chile, when physical activity typically begins to increase after its nadir in winter months [85]. Further local research is needed to understand climactic determinants of unhealthy physical fitness in Chile, given its geographical diversity. Such research will help inform public health interventions in regions with particularly high levels of unhealthy fitness.

In the Santiago Metropolitan Region, prevalence of unhealthy physical fitness was considerably higher outside of the Northeast sector (Figure 2 and Table 3). The Northeast sector of Santiago Metropolitan is the most economically developed in the region [83], suggesting, again, a potential link between higher SES and lower prevalence of unhealthy adolescent physical fitness [84]. Further investigation into the relationship between fitness and SES at the individual level in Chile is warranted. Interventions should be prioritized in areas with the highest prevalence of unhealthy physical fitness across multiple indicators, such as the North and the Southwest sectors of the city.

Considering urban vs. rural differences in the Santiago Metropolitan Region (90.5% urban), urban areas had a higher prevalence of unhealthy MSF, but a lower prevalence of unhealthy BMI and WC, compared with their rural counterparts. By contrast, outside of the Santiago Metropolitan Region, students from urban areas had a higher prevalence of unhealthy CRF compared with their rural counterparts. The higher prevalence of unhealthy CRF in urban areas could be explained, in part, by the higher prevalence of smoking in urban areas, where 43% of individuals over 15 years of age smoke, compared with 28% of their rural counterparts [27]. The other three fitness variables did not differ by urban or rural area (Table 3), an unexpected finding, as physical inactivity and obesity are higher in rural areas according to other recent Chilean national surveys [27]. More research is needed to better understand the correlates of unhealthy fitness in urban and rural areas of Chile, as studies on the topic from other countries are inconsistent [86]–[91]. A better local understanding of the influence of urbanicity on fitness may facilitate the identification of modifiable environmental factors to guide interventions and policies.

### Strengths and limitations

This study has several analytical strengths. First, the study determined the prevalence of unhealthy fitness using four health-related [3], valid [31], and reliable (with the exception of WC) [32] field tests, recommended for youth fitness assessment in the school setting [22], [23]. Low reliability in WC can be improved with training [32], suggesting reliability of WC in the present study may have been acceptable, as measurement was facilitated by a trained MINEDUC evaluator [29]. To interpret the results of these fitness tests, we used age-and-sex-specific, health-related cut-points [39], [40], [42], [46]. In addition, the study’s large sample, collected at a national level, allowed for the construction of representative fitness estimates at a regional level and the exploration of geographical variation.

There are several potential limitations in this study. First, the health-related cutoffs used, in particular for CRF and MSF, were developed in either a U.S. [39], [42], [46] or European [40] population and therefore may have limited generalizability in a Chilean population. Second, the urban or rural status for 13% of the sample could not be determined (Table S1), bringing about potential for selection bias. Among students for whom urban or rural status could not be determined, girls had higher prevalence of unhealthy CRF, and boys had higher prevalence of unhealthy MSF, BMI, and WC (Table S1), compared with their counterparts with urban or rural data. Selection bias was possible for the CRF test. In the working sample of students, 10% had missing CRF data, in large part because they were deemed ineligible after a walking screening test [29]. Students excluded from the CRF test were significantly more likely to be female and have unhealthy MSF, BMI, and WC (all p<0.05; Table S1), suggesting these students may have also had higher prevalence of unhealthy CRF. Selection bias was also possible between the 28,649 students considered for fitness evaluation and the 19,929 students for whom at least one fitness characteristic was measured, as students were excluded due to, among other reasons, temporary or chronic illness [29]. Another potential limitation is the equation used to estimate CRF [37], which may underestimate CRF by up to 12%, relative to other methods [92] and therefore may have, in isolation, inflated the prevalence of unhealthy CRF. In addition, at most, 7% of students do not matriculate in 8th grade [30], suggesting a nationally representative sample of 8th graders closely approximates a representative sample of all 8th-grade-age adolescents in the country. Probability of non-matriculation increased with decreasing income [30]. Given the potential link between lower SES and higher prevalence of unhealthy fitness [84], these data suggest that matriculating 8th-grade students may have had lower prevalence of unhealthy fitness than their non-matriculating counterparts of similar age. Considering the net effect of the mentioned potential biases, the prevalence estimates of unhealthy CRF, and to some extent other fitness variables, can be considered conservative.

### Public health implications

Low physical fitness, including poor body composition, is largely a reversible condition through appropriate lifestyle interventions, including a healthy diet, adequate PA, and, in the case of Chile, efforts to reduce adolescent smoking [93], [94]. Evidence for the effectiveness of school-based interventions to increase PA [95] and to prevent overweight and obesity [93] in adolescents in Latin America is strong. It is recommended that schools assume a leadership role in promoting youth PA and healthy lifestyles [96]. Schools are also an ideal location for sex-sensitive PA promotion to address the observed higher levels of unhealthy fitness in girls, by, for example, offering a wider range of non-competitive activities [97]. As of 2013, a series of policy changes began to occur in Chilean schools, including increased physical education from three to four hours per week in elementary schools, which is in line with the U.S. Centers for Disease Control and Prevention physical education recommendations [98]; reductions in salt and sugar and increased fruits and vegetables in school lunch programs; and better financing of sport programs, sport classes, sport infrastructure, and physical education instructors salaries [99]. These changes are a step in the right direction and should continue to be evaluated. The present study’s identification of regions in the country and sectors in Santiago with particularly high prevalence of unhealthy physical fitness can help guide the targeted implementation of such interventions aimed at improving CRF, MSF and body composition as needed locally. The inclusion of a physical education assessment in the national education survey reflects a sophisticated understanding of the importance of physical fitness for the current and future health status of Chile’s youth. This foresight allows Chile the unique opportunity to use nationally representative data to help develop and evaluate NCD primordial prevention programs in the country [63]. Chile’s experience can also help guide youth physical fitness surveillance in Latin America and in other less developed regions of the world, such as Asia, the Eastern Mediterranean, and Africa, where surveillance of this important health marker has been poor [11], but where the burden of NCDs and inactivity are rising [7], [18], and where local data are desperately needed to drive action [100].

## Supporting Information

Table S1 **Unadjusted relative prevalence of having a missing value in cardiorespiratory fitness, musculoskeletal fitness, or urban vs. rural by other demographic and fitness characteristics in the working sample (N = 19,904) of Chilean 8th-grade students: The 2011 National Physical Education Survey.**
(DOCX)
