# Supplementary material for: Geographical Variation in Health-Related Physical Fitness and Body Composition among Chilean 8th Graders: A Nationally Representative Cross-Sectional Study
Source: PLoS One. 2014 Sep 25;9(9):e108053. doi: 10.1371/journal.pone.0108053 (PMC4177890; doi:10.1371/journal.pone.0108053)
Supplement: Table S1 — Unadjusted relative prevalence of having a missing value in cardiorespiratory fitness, musculoskeletal fitness, or urban vs. rural by other demographic and fitness characteristics in the working sample (N = 19,904) of Chilean 8th-grade students: The 2011 National Physical Education Survey. (DOCX) [file pone.0108053.s001.docx]

| **Table S1.** Unadjusted relative prevalence^†^ of having a missing value in cardiorespiratory fitness,^a^ musculoskeletal fitness,^b^ or urban vs. rural^c^ by other demographic and fitness characteristics in the working sample (N=19,904)^d^ of Chilean 8th-grade students: The 2011 National Physical Education Survey | | | | | | |
| --- | --- | --- | --- | --- | --- | --- |
|  | **Missing cardiorespiratory fitness** | | | **Missing musculoskeletal fitness** | **Missing urban vs. rural** | |
|  | **(n, miss = 1,976)** | | | **(n, miss = 127)** | **(n, miss=2,636)** | |
| **Demographic characteristics** | *Boys* | *Girls* | *Both sexes* | | *Boys* | *Girls* |
| Female (ref=male) | 1.67*** | | NS | | NS | |
| Age 16-17.9 years (ref=13-15.9) | NS | NS | 10.70*** | | NS | NS |
| Low through med-high socioeconomic status^e^ (ref=high) | NS | 1.04*** | NS | | NS | 1.04* |
| Subsidized or public school (ref=private) | NS | 1.04*** | 1.06** | | NS | 1.03* |
| Urban^c^ (ref=rural) | 1.20*** | 1.16** | NS | | n/a | n/a |
| Santiago Metropolitan (ref=else) | 1.32** | 1.37*** | NS | | 0^‡^*** | 0^‡^*** |
|  |  |  |  | |  |  |
| **Health-related fitness** |  |  |  | |  |  |
| Unhealthy cardiorespiratory fitness^a^ (ref=healthy) | n/a | n/a | NS | | 1.43*** | 1.10* |
| Unhealthy musculoskeletal fitness^b^ (ref=healthy) | 1.74*** | 1.40*** | n/a | | 1.16* | NS |
| Unhealthy body mass index^f^ (ref=healthy) | 1.49*** | 1.29*** | NS | | 1.16*** | NS |
| Unhealthy waist circumference^g^  (ref=healthy) | 1.62*** | 1.33*** | NS | | 1.14* | NS |
| ^†^Values shown are relative prevalence of a missing value compared to reference group. Musculoskeletal fitness missing values were not stratified by sex due to the relatively low number of missing values. Prevalence ratios are only shown if comparison was significant at α=0.05. Abbreviations: ref, reference group; miss, missing; NS, not significant at α=0.05; n/a, not applicable; ^a^Unhealthy cardiorespiratory fitness is combined needs improvement and needs improvement – health risk FITNESGRAM 2011 maximal aerobic capacity groups [[39](#_ENREF_39)]. ^b^Unhealthy musculoskeletal fitness is standing broad jump below the 20th percentile of European adolescents [[14](#_ENREF_14)]. ^c^Berdegue et al [[47](#_ENREF_47)]. ^d^Working sample excludes individuals 18 years and older or with missing BMI. ^e^Groups defined by Chilean Ministry of Education [[101](#_ENREF_101)].^f^Unhealthy body mass index is combined needs improvement and needs improvement – health risk FITNESGRAM 2011 BMI groups [[42](#_ENREF_42)]. ^g^Waist circumference is classified according to health-related cut-points [[46](#_ENREF_46)]. ^‡^All missing values in urban vs. rural are outside Santiago Metropolitan Region.***p<0.001; **p<0.01; *p<0.05 | | | | | | |
